# A Manual of Obstetrics

**Published:** 1885-01

**Authors:** 


A Manual of Obstetrics—By Edward L. Partridge, M. D., Professor of Obstetrics, New York; Post Graduate Medical School; Instructor in Obstetrics, College of Physicians and Surgeons, New York; Visiting Physician to the Maternity Hospital, and to the Nursery and Child's Hospital; Attending Gynecologist to the New York Hospital, out-patient Department; Fellow of the New York Obstetrical Society; with sixty illustrations, New York: William Wood & Co., 1884.

If the above titles of the author do not suffice to create a presumption in favor of his competency to instruct others, these statements in his preface should define his status: "A considerable experience in class teaching has permitted the writer to know what subjects are stumbling blocks to the student." "The writer having also a large personal experience in obstetrics, both in hospital and private practice, believes himself aware of the needs of the practitioner as well."

With such assurances of practical discernment, we have sought to understand the lessons presented in this little volume, and trust that no misconception of "that obstetric knowledge, especially called for on the part of the medical student and general practitioner," will mark the comments which its careful perusal suggests. The mere routine injunctions which are applicable to ordinary cases of labor are for the most part satisfactory, but whenever anything extraordinary and demanding interference is referred to, the light imparted to guide the obstetrician does not always so illumine his path as we should expect from such a great luminary. Among other noticeable short comings are those in regard to the management of "antepartem hour-glass contraction of the uterus," and post-partem "irregular uterine contraction"

Had not our author put forth a claim, not only to knowledge of the subject, but to practical skill, it might be inferred that he was not acquainted with Fancourt Barnes' use of the nitrite of amyl in this class of cases, nor of the extreme anesthesia of chloroform, by which relaxation of the uterine fibres is effected; to say nothing of the safe and efficient manual dilatation of these rigid cord-like contractions of the womb upon the fœtus or upon the placenta.

How it would be practicable without the above means, to accomplish any good result by forceps or embryotomy, in that "tetanoid contraction of bundles of transverse, or of oblique uterine fibres, which grasp and retain the fœtus in utero," is incomprehensible.

Again, in treating of "transverse presentations," it is a complete begging of the question when he says, "If the child in its mal-presentation cannot be dislodged from the pelvis, embryulcia with, in some instances decapitation, will be required."

With the lights before obstetricians at the present day as to the practicability of so relaxing the womb by profound anesthesia as to render a correction of these positions simple and easy, no one would be warranted in practicing decapitation of a child.

In referring to the modes of proceeding with adherent placenta, no allusion is made to one of the most efficient measures by the injection of cold water through the cord; but great stress is properly laid upon its prompt removal by the hand, and "at the same time grasp the uterus through the abdomen with the other hand, and compel it to descend and contract." It is an instance of over-caution to enjoin in cases of natural labor that "after the placenta is expelled, pressure and gentle manipulation of the uterus should be kept up for one-half to three-quarters of an hour."

While noting these exceptions to the general rule of trustworthiness, this manual of obstetrics is calculated to be very useful.

